# How Does Adult Attachment Affect Human Recognition of Love-related and Sex-related Stimuli: An ERP Study

**DOI:** 10.3389/fpsyg.2016.00596

**Published:** 2016-05-02

**Authors:** Juan Hou, Xin Chen, Jinqun Liu, Fangshu Yao, Jiani Huang, Yamikani Ndasauka, Ru Ma, Yuting Zhang, Jing Lan, Lu Liu, Xiaoyi Fang

**Affiliations:** ^1^Department of Philosophy, Anhui UniversityHefei, China; ^2^School of Humanities and Social Science, University of Science and Technology of ChinaHefei, China; ^3^CAS Key Laboratory of Brain Function and Disease, School of Life Science, University of Science and Technology of ChinaHefei, China; ^4^Institute of Developmental Psychology, Beijing Normal UniversityBeijing, China

**Keywords:** adult attachment, love, sex, event-related potentials (ERPs), emotion-motivation system

## Abstract

In the present study, we investigated the relationship among three emotion-motivation systems (adult attachment, romantic love, and sex). We recorded event-related potentials in 37 healthy volunteers who had experienced romantic love while they viewed SEX, LOVE, FRIEND, SPORT, and NEUTRAL images. We also measured adult attachment styles, level of passionate love and sexual attitudes. As expected, results showed that, firstly, response to love-related image-stimuli and sex-related image-stimuli on the electrophysiological data significantly different on N1, N2, and positive slow wave (PSW) components. Secondly, the different adult attachment styles affected individuals’ recognition processing in response to love-related and sex-related images, especially, to sex-related images. Further analysis showed that voltages elicited by fearful attachment style individuals were significantly lower than voltages elicited by secure and dismissing attachment style individuals on sex-related images at frontal sites, on N1 and N2 components. Thirdly, from behavior data, we found that adult attachment styles were not significantly related to any dimension of sexual attitudes but were significantly related to passionate love scale (PLS) total points. Thus, the behavior results were not in line with the electrophysiological results. The present study proved that adult attachment styles might mediate individuals’ lust and attraction systems.

## Introduction

Sexual relationship, in the context of human mating, plays an important role in the development as well as the evolution of human beings. There are three primary emotion-motivation systems in human brain during the development of human sexual relationship for mating, reproduction and parenting: lust, attraction, and attachment (Fisher, 1998). Sex drive (also called the libido or lust) is regarded as a craving for sexual gratification while romantic love (attraction, obsessive love, or passionate love) is considered as a powerful energy, feeling of exhilaration and focused attention on someone who catches our attention (Fisher et al., 2002). Attachment, previously regarded only as intensive bond between mother and infant (Bowlby, 1969/1982), now includes adult male-female attachment and is divided into three types, namely, secure, avoidant, and anxious (Hazan and Shaver, 1987). One of the most difficult dilemmas in relationship science is the interaction between sexual desire and love (Hatfield and Rapson, 1993; Regan and Berscheid, 1999; Diamond, 2004). Our study seeks to assess whether adult attachment could mediate recognition processing of love and sexual desire.

Love involves complex neural mechanisms and is a result of chemical, cognitive and directed behavioral components (Aron and Aron, 1986, 1996; Bartels and Zeki, 2000, 2004; Mashek et al., 2000; Buss, 2003; Aron et al., 2005; Esch and Stefano, 2005; Fisher et al., 2005). A study of relevance found that when individuals viewed pictures of their loved ones, the activity restricted foci in the medial insula and the anterior cingulate cortex, the caudate nucleus, and the putamen (Bartels and Zeki, 2000). A functional Magnetic Resonance Imaging (fMRI) study verified the hypothesis that love is a goal-directed state that leads to a range of emotions, rather than a specific emotion (Aron et al., 2005). Another study found that subliminal presentation of either a beloved’s name (love prime) or a passion descriptor (passion prime) enhance reaction times in a similar fashion (Ortigue et al., 2007). These studies showed that, compared to friendship, romantic love is associated with mediating reward, emotion, and motivation systems (Bartels and Zeki, 2000, 2004; Aron et al., 2005; Fisher et al., 2005; Ortigue et al., 2007). Another study found that regional brain activity during early stage intense romantic love predicted relationship outcomes after 40 months (Xu et al., 2012). By using event-related potentials (ERPs), another study found that when participants viewed faces of their beloved, friend and unknown but beautiful people, the late positive potential (LPP) was larger in reaction to the face of the beloved than the other two types of faces (Langeslag et al., 2007). This entails that romantic love enhances one’s lasting attention to faces of his/her beloved.

Different from romantic love, which is described as a delighted feeling or craving for established emotional union with preferred partner, sexual desire is defined as a motivation to have sex with any appropriate partners (Fisher, 2004). Sexual arousal in humans is a complex experience, which includes both physiological and psychological processes. Brain activity plays a crucial role in sexual desire. Researchers use fMRI and positron emission tomography (PET), to investigate regions of brain associated with erotic stimuli. These techniques provide high spatial resolution, allowing researchers to study brain regions at a very macroscopic level (Stoléru et al., 1999; Redouté et al., 2000; Bocher et al., 2001; Karama et al., 2002; Mouras et al., 2003; Ferretti et al., 2005). Three PET studies used ^15^O-H_2_O to measure responses of regional cerebral blood flow (rCBF) in healthy males elicited by visual erotic film clips. After comparing them with other neutral control clips, the studies found that sexual stimuli were related to activation of some paralimbic areas (like anterior cingulate gyrus, orbitofrontal cortex, right insula and right inferior frontal cortex (Stoléru et al., 1999; Redouté et al., 2000; Bocher et al., 2001). Mouras et al. (2003) found that sexual stimuli were associated with increased activation of the parietal lobes, the right parietooccipital sulcus, the left superior occipital gyrus and the precentral gyri. Another study investigated the effect of emotional valence and arousal value of non-erotic visual stimuli on ERPs and found that positive valence and high arousal resulted in larger P300 and PSW (Lankveld and Smulders, 2008). Based on these results, we can rightly consider sexual arousal as a composite psychophysiological state correlated with activation/deactivation of several brain regions including cognitive, emotional, motivational, and autonomic components (Redouté et al., 2000).

The attachment theory generated by Bowlby (1969/1982) was conceived after observing separation between infants and their primary caregivers. The study found that infants’ reaction could be predictable in three ways- protest, despair and detachment. After including adult male-female attachment the three ways have been re-termed as- secure, avoidant, and anxious, indicating that attachment is not only limited to childhood but also could be translated to romantic love process (Hazan and Shaver, 1987). Bartholomew and Horowitz (1991) proposed a creative 4-group model of adult attachment style- secure, preoccupied, dismissing and fearful. Compared to insecure attachment, secure individuals are much more easily engaged in stable relationships (Hazan and Shaver, 1987; Kirkpatrick and Hazan, 1994). Feeney and Noller (1990) reported that adult attachment was intensively associated with various forms of love and beliefs of relationship. Another study reported that avoidant people could mistrust others and engage in less intensive romantic love (Sepah-Mansour et al., 2009). Ahmadi et al. (2013) reported that ambivalent attachment style could significantly predict obsessive love. Attachment style was reliable in predicting some sexual attitudes and behavior, especially anxiety attachment, which predicted sexual attitude in late adolescence (Feeney et al., 2000). Gentzler and Kerns (2004) also found that insecure attachment people had links with some sexual experience. For instance, avoidant attachment was associated with engagement in casual sex and anxious attachment was related to more unwanted but consensual sexual experiences. Birnbaum (2007) found that all attachment orientations were related to aversive sexual affect and cognitions within romantic relationships. Different adult attachment is also related to perception of emotional-laden stimuli, especially when the stimuli contain social information (Vrtička et al., 2012). From the above, we can conclude that adult attachment may affect various forms of love and sexual attitudes.

As mentioned above, the processing of romantic love stimuli and sexual stimuli were generally depicted by using fMRI and PET in the human sexual relationship, which involved the high spatial resolution but were limited in the temporal resolution. It is obvious that the limited of temporal resolution cannot measure the early stage of processing at the precise millisecond level. The present study aimed to use the method of ERPs, which have the high temporal resolution and could be time-locked to the onset of different categories stimuli. Therefore, ERP-measurement is suitable for measuring the early stage processing features of love-related stimuli and sex-related stimuli.

The first purpose of this study was to investigate ERPs in response to love-related images and sex-related images using electrophysiological data. Secondly, we also tested whether adult attachment styles affect one’s recognition processing in response to love-related images and sex-related images. Thirdly, based on behavioral data, we tested whether individuals with different attachment styles show significant difference in levels of passionate love and sexual attitudes. Taking into consideration previous studies, high temporal resolution of ERPs would provide a valuable tool to investigate early stages of processing love-related stimuli and sex-related stimuli. Our study is the first to simultaneously investigate three primary emotion-motivation systems in human’s brain- lust, romantic love and attachment, by using high temporal resolution ERP technology. We tested the following hypotheses: H1- response to love-related images will significantly differ from response to sex-related images on electrophysiological data. H2- different adult attachment styles will affect one’s recognition processing in response to love-related images and sex-related images. H3- adult attachment style will affect individual’s passionate love level and sexual attitudes based on behavior data as well as electrophysiological data.

## Materials and Methods

### Participants

Participants were recruited using a poster at the university campus and on the Internet. Forty-five undergraduate and graduate students at Anhui University in China volunteered to participate in this study. Finally, 8 invalid data were discarded because of artifact rejection from EEG while 37 valid data remained for further analysis. The remaining 37 participants (17 males, 20 females; mean age 22.8, range 20–32) were all heterosexual and reported to either being in love or to have previously been in love using the questionnaire like. All participants were healthy, with normal or corrected-to-normal vision, no history of neurological or psychiatric disorder. Furthermore, all 37 participants were right-handed. Each participant signed a written informed consent before taking part in the experiment and received 50 RMB (approximately US $8) as compensation.

### Ethics Statement

Each participant to the study provided written informed consent after receiving an explanation of the study’s purpose and procedure. The study was approved by the Human Research Ethics Committee of Anhui University of China according to the principles expressed in the Declaration of Helsinki. Participants were undergraduate and postgraduate students. We did not obtain informed consent from guardians of participants whose age was under 18. These young college students were considered to have comparable intelligence and ability to adult students, and able to take charge of their behaviors. According to the General principles of the Civil Law of the People’s Republic of China; “A minor aged 10 or over shall be a person with limited capacity for civil conduct and may engage in civil activities appropriate to his age and intellect; in other civil activities, he shall be represented by his agent ad litem or participate with the consent of his agent ad litem” (Article 12, Chapter II).

### Measures

Attachment to romantic partners: participants completed the Chinese version of Experiences in Close Relationships (ECR) Questionnaire (Li and Kato, 2006). This 36-item questionnaire includes 18 items measuring avoidance attachment, the other 18 items measuring anxiety attachment about romantic relationships. Each item was rated on a 7-point Likert scale (ranging from 1 = strongly disagree to 7 = strongly agree). The Cronbach’s alphas for the avoidance and anxiety scales were high (0.832 for both scales). According to the method of Griffin and Bartholomew (1994a,b), we divided the participants into four attachment styles groups (secure, preoccupied, dismissing and fearful).

Passionate love level: in order to test this, we used the short version of PLS, developed by Hatfield and Sprecher (1986). This scale consists of 30 items and each item is rated on a 9-point Likert scale (ranging from 1 = strongly disagree to 9 = strongly agree), higher scores indicating higher levels of love. The Cronbach’s alpha of this scale was 0.857.

Sexual attitude: participants completed the Brief Sexual Attitudes Scale (BSAS), which has four subscales of Permissiveness, Birth Control (formerly called Sexual Practices), Communion, and Instrumentality. This scale includes 23 items and each item is rated on a 5-point Likert scale (ranging from 1 = strongly disagree to 5 = strongly agree). Higher scores indicated higher open sexual attitude. The Cronbach’s alpha of this total scale was 0.851.

We engaged two colleagues to translate the above two scales from English to Chinese and engaged another pair of colleagues to translate it back to English. We compared the translations and came up with the best translation.

### Materials

Materials for the experiment included video clips and visual images. We obtained three different copyright-free video clips (erotic, non-erotic love, and friendship) from publicly shared online videos. Erotic video clip showed heterosexual couples engaged in intercourse, non-erotic love video clip showed heterosexual couples in romantic love without any sexual behavior and friendship video clip showed male-female friendship. Each video clip lasted for not more than 6 min and all participants used earphones to listen to the sound of the videos during the experiment. We used the video clips as cues for image-stimuli that followed and were not analyzed in detail. The three types of video clips were used for creating three different states and making subjects a better understanding of the followed three image-stimuli (erotic, non-erotic love and friendship).

As mentioned in the introduction, previous related ERPs studies used faces of beloved or friends, erotic sex pictures, sports pictures, and neutral pictures as experimental materials (Langeslag et al., 2007; Lankveld and Smulders, 2008). The previous love-related ERP studies chose the love-related and friend-related facial stimuli as the experimental materials for control some other confounded variables such as familiarity and perceived beauty (Langeslag et al., 2007). As to the sex-related study, researchers chose the sport stimuli as the contrast stimuli for separating the ERP-contributions of erotic content from that of arousal value and hedonic valence, that is the erotic picture was equivalent to the category of high-energy sports pictures on the dimensions of valence and arousal (Lankveld and Smulders, 2008). In order to compare love-related stimuli and sex-related stimuli simultaneously, we divided the original visual images of our study into five categories. (1) 20 SEX images (nude heterosexual couples engaging in intercourse while showing female breasts, but without showing female or male genital areas in close-up). (2) 20 LOVE images (heterosexual couples dating, kissing and hugging without any erotic behavior). (3) 20 FRIEND images (friends of opposite sex talking and playing with no physical contact). (4) 20 SPORT images (fencing, kickboxing, skiing, and running) and (5) 20 NEUTRAL images (beautiful scenery like forests, mountains, and rivers). Images from the first three categories (LOVE, FRIEND, and SEX) were taken from erotic, non-erotic love and friendship video clips (as mentioned in the above paragraph) respectively, while images from the other two categories (SPORT and NEUTRAL) were downloaded from free sites on the Internet. We chose the SPORT images to match possible motion factors in the SEX images during the ERP experiment whilst FRIEND images were chosen to match possible friendship factors in the LOVE images during the ERP experiment. Each image depicted people’s behavior only and contained two opposite sex persons of Asian origin. All images were standardized for brightness, saturation and size (600 pixels × 450 pixels) with Photoshop CS4. Before the experiment began, eight males and eight females, not involved in the study, independently rated these images on levels of quality (1-extremely bad to 7-extremely good), sexual arousal (1- no arousal to 7- highest arousal level) and love arousal (1- no arousal to 7-highest arousal level). According to their mean scores, 40 images were selected for the formal experiment, each category included eight images. In addition, the center of each image had a cross-shaped image (65 pixels × 65 pixels; white background and black foreground) which contained two shapes in which horizontal line was longer than vertical line or vertical line was longer than horizontal line. Two cross-shaped images occurred in the five categories randomly. When the shape changed, participants were instructed to press “1” on the keyboard, using the right hand. The task was used to keep participants focused on the images.

### Procedure

After arriving at the laboratory, participants read and signed an informed consent form, which included a brief description of the procedure. Participants then filled in questionnaires and provided information about the duration of their recent romantic relationships as well as other demographic variables. Next, we placed electrodes on participants’ heads and instructed them to limit movements and eye blinks during experiment. The participants sat in a comfortable chair in a soundproof, dimly lit room and put on their earphones. Images were displayed on a 49-cm monitor, with a maximum size of 27 cm × 37 cm, presented approximately 1.25 m from the participant’s eyes with a visual angle of 16° horizontally and 12° vertically. Stimuli were presented in two blocks of 24 images each and each block contained 8 images from three different categories. One block called LOVE was about love, and the other block called SEX was about sex. The order of two blocks was counter-balanced across participants to control for any sequence or carryover effects. Stimuli of the same categories appeared in random order within each block. Stimuli presentation was controlled by E-Prime software.

Before each block, we showed participants a video clip (erotic; before the SEX block) or two video clips (friendship and non-erotic love; before the LOVE block) to make participants understand the relationship between the two persons of the following stimuli, which could render the love-related or sexual-related emotions. We did not analyze the recorded EEG during the video time. There was a 1-min break after video clip(s). Each block started with a black fixation cross (5 cm × 5 cm) in the center of the white computer screen and lasted for 500 ms. Then stimuli were presented for 350 ms. Specifically, the stimuli were presented in the order of FRIEND-NEUTRAL-LOVE (in LOVE block) or SPORT-NEUTRAL-SEX (in SEX block). Images in the same categories appeared in random order within each block. Each category (SEX, LOVE, FRIEND, SPORT, and NEUTRAL) contained eight images and each image was presented 50 times in one block, thus, the study consisted 2400 trials. Furthermore, once participants found the cross-shaped image in the center of each stimuli and pressed “1” on the keyboard using the right hand, the shape changed. There were several times for resting during the experiment.

### Electroencephalogram (EEG) Recording and Signal Processing

The electroencephalogram (EEG) was recorded using a 64-channel amplifier (SynAmps 2, Neuroscan) and data acquisition software (SCAN4.3, Neuroscan). The 64 Ag–AgCl active electrodes were placed on the scalp by means of a head cap, according to the 10–20 International System. Scalp impedance for each electrode was kept below 5 kΩ. Vertical electro-oculogram (VEOG) was recorded by attaching additional electrodes above and below the left eye. The REF electrode served as reference and the forehead GND electrode was used as ground. All signals were digitized with a sample rate of 500 Hz, a 24-bit A/D conversion and a 0.05–100 Hz band pass filter.

The offline analysis of ERP data was performed with Neuroscan 4.3 software. Data were filtered using a band pass filter of 0.5–25 Hz with zero phase shifts (24 dB/octave slope). Ocular artifact correction was applied according to the Gratton and Coles algorithm. Data epochs were extracted from a time window between 200 ms before and 750 ms after the stimuli onset. The mean 200 ms pre-stimuli period was used for baseline correction. Artifact rejection criteria were minimum and maximum baseline-to-peak -50 to +50 μV. According to previous studies and the purpose of current study, average ERPs were then computed for each participant and the five images categories (SEX, LOVE, NEUTRAL, SPORT, and FRIEND). The following nine electrode positions were analyzed: F3, Fz, F4, C3, Cz, C4, P3, Pz, and P4 (cf. Langeslag et al., 2007; Lankveld and Smulders, 2008). The voltages and waves that were elicited by SEX, LOVE, SPORT and FRIEND stimuli images during the experiment were recorded.

### Analysis

The questionnaire and ERP data were analyzed using SPSS 11.0 for Windows, the mean ERP voltages in the time windows 100–200 ms (referred to as N1 component), 200–300 ms (referred to as N2 component), and 500–750 ms (referred to as PSW component) were tested at several electrodes (frontal: F3, Fz, F4; central: C3, Cz, C4; parietal: P3, Pz, P4). The considered two difference waves were created through the SEX minus SPORT (referred to as SEX-MINUS-SPORT wave) and LOVE minus FRIEND (referred to as LOVE-MINUS-FRIEND wave). The minus waves were created for further analysis. Using repeated measures ANOVAs with the within-subject factors categories (SEX-MINUS-SPORT, LOVE-MINUS-FRIEND) and nine electrodes (frontal: F3, Fz, F4; central: C3, Cz, C4; parietal: P3, Pz, P4), and with N1, N2, and PSW as dependent variables. The interaction effects between difference waves (SEX-MINUS-SPORT wave, LOVE-MINUS-FRIEND wave) and the between-subject factors four attachment styles (secure, preoccupied, dismissing, and fearful) were tested using the method of repeated measures ANOVAs. Only effects involving the factor condition of interest were reported. Greenhouse-Geisser correction for violations of the sphericity assumption in repeated measures analyses was used when appropriate. Effects were considered significant when *p* < 0.05. Significant interaction effects were followed-up by paired-samples *t*-tests.

## Results

### Electrophysiological Data

**FIGURE 1:**
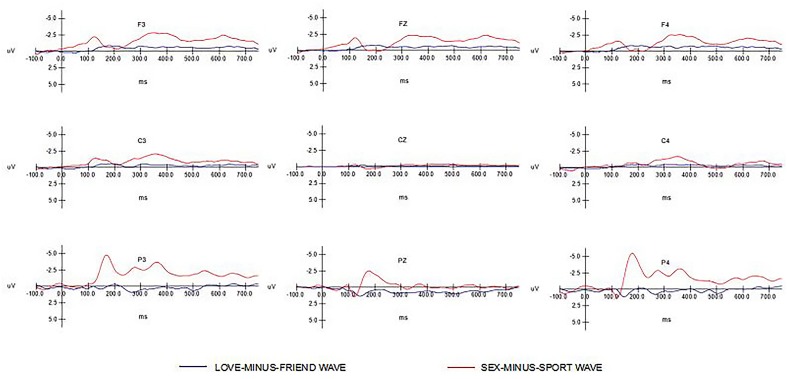
**Grand-average (*N* = 37) event-related potentials (ERPs) at various electrode sides as a function of difference waveforms types (SEX MINUS SPORT wave, LOVE MINUS FRIEND wave)**.

**FIGURE 2:**
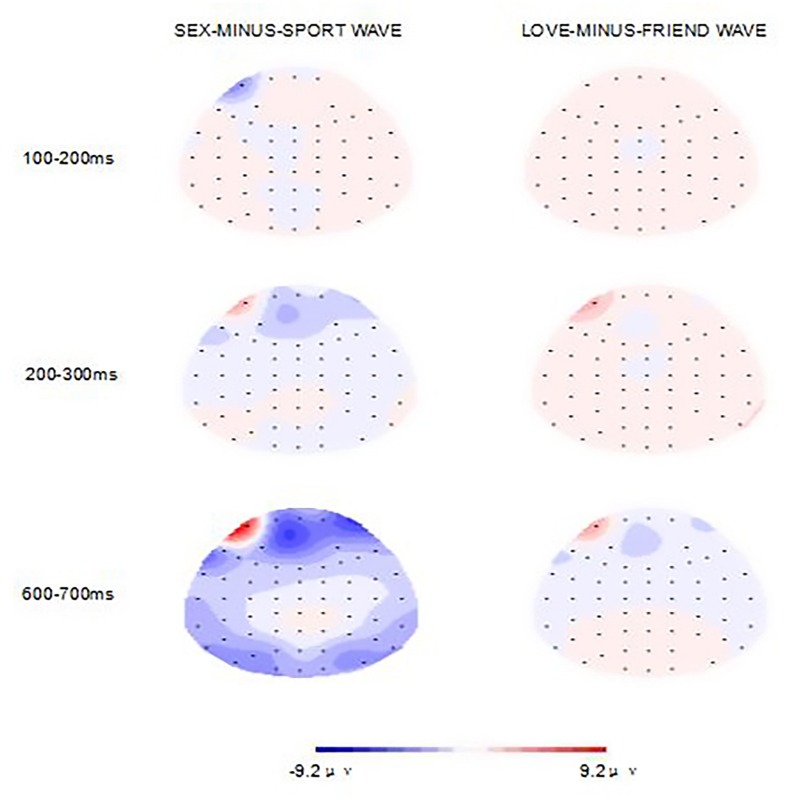
**Voltage scalp distributions for the two difference waveforms between 100 and 200 ms, 200 and 300 ms, and 600 and 700 ms after image-stimuli onset**.

Dependent variables were area measures under the two ERP difference waveforms (SEX-MINUS-SPORT wave, LOVE-MINUS-FRIEND wave) in the latency windows of interest. **Figure 1** shows the grand average waveforms per difference image stimuli at frontal (F3, Fz, F4), central (C3, Cz, C4), and parietal (P3, Pz, P4) electrode sites. Apparently, the amplitude of SEX-MINUS-SPORT wave was larger than the LOVE-MINUS-FRIEND wave especially at parietal. **Figure 2** shows the voltage scalp distributions for the two minus-difference waveforms between 100 and 200 ms, 200 and 300 ms, and 500 and 750 ms after image-stimuli onset.

#### N1 (100–200 ms)

A 2 (SEX MINUS SPORT wave, LOVE MINUS FRIEND wave) × 9 (electrodes) repeated within-subjects ANOVA was tested on this time window (100–200 ms). Both the main effect of two difference waves [*F*(1,36) = 31.30, *p* < 0.00] and nine electrodes [*F*(8,288) = 7.236, *p* < 0.00] were significant. The interaction between stimuli categories and electrode was also significant [*F*(8,288) = 11.972, *p* < 0.00].

#### N2 (200–300 ms)

A 2 (SEX MINUS SPORT wave, LOVE MINUS FRIEND wave) × 9 (electrodes) repeated within-subjects ANOVA was tested on this time window (200–300 ms). Both the main effect of two difference waves [*F*(1,36) = 50.25, *p* < 0.00] and nine electrodes [*F*(8,288) = 9.96, *p* < 0.00] were significant. The interaction between stimuli categories and electrode was also significant [*F*(8,288) = 40.58, *p* < 0.00].

#### PSW (500–750 ms)

A 2 (SEX MINUS SPORT wave, LOVE MINUS FRIEND wave) × 9 (electrodes) repeated within- subjects ANOVA was tested on this time window (500–750 ms). Both the main effect of two difference waves [*F*(1,36) = 39.42, *p* < 0.00] and nine electrodes [*F*(8,288) = 18.67, *p* < 0.00] were significant. The interaction between stimuli categories and electrode was also significant [*F*(8,288) = 10.11, *p* < 0.00].

### Effects of Image Stimuli Categories and Attachment Style

According to the ECR Questionnaire, we divided participants into four attachment style groups- secure (*n* = 7), fearful (*n* = 17), preoccupied (*n* = 9), and dismissing (*n* = 4).

We performed a two-way repeated measure ANOVA to test whether four adult attachment styles (secure, preoccupied, dismissing, and fearful) could have interaction effects with the grand average voltages of SEX-MINUS-SPORT wave and LOVE-MINUS-FRIEND wave on N1, N2, and PSW components at each electrode. The interaction effects results are shown in **Table 1.** Results showed that adult attachment styles had significant interaction effects with difference waves at electrodes (F3, FZ, and F4) on N1 and N2 components, at electrodes (CZ, C4, and PZ) on N2 component, at electrode C4 on PSW component.

**Table 1:** Interaction effects between difference wave (SEX-MINUS-SPORT wave, LOVE-MINUS-FRIEND wave) and adult attachment on N1, N2, and positive slow wave (PSW) per electrode.

Electrodes	N1 (100–200 ms)	N2 (200–300ms)	PSW (500–750ms)
F3	3.057^∗^	3.658^∗^	1.123
FZ	3.775^∗^	4.461^∗∗^	1.405
F4	4.106^∗^	4.731^∗∗^	2.417
C3	0.765	0.212	0.687
CZ	1.855	3.630^∗^	0.289
C4	2.200	5.939^∗∗^	4.282^∗^
P3	2.311	0.487	0.459
PZ	1.403	3.200^∗^	0.306
P4	1.634	0.902	0.663


*^∗^*p* < 0.05, ^∗∗^*p* < 0.01.*

We further tested multiple comparisons in order to understand specific differences among the four adult attachment styles at the above significant electrodes on N1. We found that voltages that were elicited by secure attachment style individuals were larger than those elicited by fearful attachment style individuals on SEX-MINUS-SPORT wave at electrodes F3 (*p* < 0.05) and F4 (*p* < 0.01). Voltages elicited by secure attachment style individuals were larger than those elicited by preoccupied attachment style individuals on SEX-MINUS-SPORT wave at electrode F4 (*p* < 0.05). Voltages elicited by dismissing attachment style individuals were larger than those elicited by preoccupied attachment style individuals on SEX-MINUS-SPORT wave at electrode F4 (*p* < 0.05).

We also tested multiple comparisons to assess differences among the four adult attachment styles at the above significant electrodes on N2. We found that voltages elicited by secure attachment style individuals were larger than those elicited by fearful attachment style individuals on SEX-MINUS-SPORT wave at electrodes F3 (*p* < 0.01), FZ (*p* < 0.01), and C4 (*p* < 0.01). Voltages elicited by secure attachment style individuals were larger than voltages elicited by preoccupied attachment style individuals on SEX-MINUS-SPORT wave at electrode FZ (*p* < 0.05), F4 (*p* < 0.01) and CZ (*p* < 0.05). Voltages elicited by dismissing attachment style individuals were larger than voltages elicited by fearful attachment style individuals on SEX-MINUS-SPORT wave at electrode FZ (*p* < 0.05), F4 (*p* < 0.05), and C4 (*p* < 0.05). Voltages elicited by dismissing attachment style individuals were larger than both preoccupied and fearful attachment style individuals on LOVE-MINUS-FRIEND wave at electrode FZ (*p* < 0.05).

In summary, our results showed that adult attachment styles are related to recognition of different stimuli (SEX or LOVE) especially at frontal sites on N1 and N2 ERP components. Further comparisons showed that voltages elicited by secure attachment style individuals are significantly larger than voltages elicited by fearful and preoccupied attachment style individuals on SEX-MINUS-SPORT wave, at frontal sites, on N1 and N2 components. Finally, voltages elicited by dismissing attachment style individuals are larger than voltages elicited by fearful attachment style individuals on SEX-MINUS-SPORT wave at frontal sites, on N2 components.

### Behavior Data and Electrophysiological Data

We used One-Way ANOVA analyses to test whether the four adult attachment styles (secure, preoccupied, dismissing, and fearful) are significantly different among between love-related and sexual-related behavior variables (i.e., PLS total points, sexual attitude dimensions: permissiveness, birth control, communion, and instrumentality). Results showed that the four adult attachment styles were significantly different in PLS and total points [*F*(3,33) = 3.34, *p* < 0.05]. LSD *post hoc* tests showed that PLS scores of preoccupied attachment style individuals were significantly higher than scores of secure attachment style individuals (*M*_D_ = 22.93, *p* < 0.01) and fearful attachment style individuals (*M*_D_ = 13.29, *p* < 0.05). See **Table 2** for detailed results.

**Table 2:** Result of one-way ANOVA of adult attachment styles, PLS total points and sexual attitudes.

	Secure (*n* = 7)	Fearful (*n* = 17)	Preoccupied (*n* = 9)	Dismissing (*n* = 4)	
	
	M ± SD	M ± SD	M ± SD	M ± SD	*F*
PLS total	85.71 ± 7.97	94.71 ± 16.56	107.11 ± 11.69	90.75 ± 15.00	3.34^∗^
Permissiveness	4.03 ± 0.58	3.61 ± 0.86	4.00 ± 0.84	3.55 ± 0.44	0.88
Birth control	1.62 ± 0.95	1.80 ± 1.00	1.63 ± 0.63	1.50 ± 0.58	0.19
Communion	1.77 ± 0.26	2.39 ± 0.17	1.89 ± 0.23	2.40 ± 0.35	1.98
Instrumentality	3.26 ± 0.40	3.01 ± 0.76	3.11 ± 0.45	2.45 ± 0.66	1.48


*^∗^*p* < 0.05.*

Results from electrophysiological data showed that adult attachment styles were related to recognition of different stimuli (SEX or LOVE) especially at frontal sites on the N1 and N2 ERP components. Further, electrophysiological voltage differences of adult attachment styles were more significant on the recognition of sex-related stimuli images, while behavior data found that adult attachment styles were not significantly related to all dimensions of sexual attitudes. Thus, results from behavior data were different from electrophysiological data.

## Discussion

In the present study, we found some interesting results. Firstly, based on our electrophysiological data, response to love-related image-stimuli and sex-related image-stimuli significantly differed on N1, N2, and PSW components. Secondly, different adult attachment styles significantly affected individuals’ recognition processing in response to love-related images and sex-related images, especially response to sex-related image-stimuli. Thirdly, adult attachment styles were significantly different in passionate love.

As discussed in the introduction, we tested attachment style, level of passionate love and sexual attitudes to understand mediation effect of adult attachment style on individuals’ processing of love-related variable (passionate love level) and sex-related variable (sexual attitudes). Our results showed that the four adult attachment styles (secure, preoccupied, dismissing, and fearful) were significantly different in PLS total points. Further analysis showed that preoccupied attachment style was significantly higher than secure and fearful attachment styles in PLS total points. This implies that preoccupied attachment individuals are extremely passionate in romantic relationships (Hatfield and Sprecher, 1986). Bartholomew and Horowitz (1991) reported that preoccupied attachment individuals are associated with more expressiveness and less coldness. Further, ambivalent attachment style can significantly predict obsessive love (Ahmadi et al., 2013). Thus, attachment style can influence individuals’ performance in sexual relationship, particularly, unsecure attachment individuals are considered more likely to fall in obsessive love.

As shown in **Figure 1**, we found that N1 (from 100 ms after stimuli onset) was more pronounced in sex-related stimuli than love-related stimuli at frontal and parietal sites. This suggests that cortical neural response was activated more by sex-related stimuli than by love-related stimuli. In the present study, sexual-related stimuli induced lager N1 components than the other categories, which, just like other emotional expressions, gets prior and more attention at early stages of cognition processing. Sex-related pictures are not only considered as one category of emotional pictures but also as a biological stimulus (Anokhin et al., 2006), which are crucial for human survival and reproduction. This helps us better understand why brain activities pay more attention to sex-related stimuli than love-related stimuli. Evolutionary and biological information from visual scenes may hence affect human brain mechanism of processing these relevant visual stimuli. Such brain mechanism could preferentially discriminate sex-related stimuli from stimuli from other categories in early stage processing. Early ERP components contain top-down regulation of sensory processing (Foxe and Simpson, 2002). These brain activities may modulate the processing in order to facilitate discerning information of biological stimuli.

Between 200 and 300 ms after stimuli onset, N2 was less pronounced in sex-related stimuli than love-related stimuli and neutral stimuli at three prefrontal electrodes. The ERP components included N1, P2, N2, and P3. N1 and P2 are regarded as early components of brain processes in response to stimuli, relying on physical properties of stimuli (strength, type, frequency, etc.), which are called exogenous components. N2 and P3 are related to human perception, recognition, attention and memory, named endogenous components. Thus, current finding of a decreased N2 for sex-related stimuli may be due to physical properties of stimuli. Besides, N2 has been found to be smaller for emotional facial stimuli than neutral facial stimuli at prefrontal electrodes (Eimer and Holmes, 2002, 2007). As such, sexual-related stimuli may be a special emotional category, which could influence this early component. Discriminative response to different picture categories starts in anterior regions of the scalp, suggesting that an involvement of the prefrontal cortex in the discrimination of specific contents.

As expected, we found that PSW (between 500 and 750 ms after stimuli onset) was lager for sex-related stimuli than love-related stimuli and neutral stimuli at frontal and parietal sites. This finding is in line with earlier studies, both in females and males (Anokhin et al., 2006). Van-Lankveld and Smulders (2008) compared difference among males in response to erotic pictures and sport pictures and found that erotic pictures induced lager P300 and PSW components. PSW is treated as the index of attention maintenance (Bradley, 2000; Schupp et al., 2003), which meanings need much more attention materials. Anokhin et al. (2006) investigated difference in neuroelectric response between erotic pictures and other picture categories and found that emotionally arousing pictures, regardless of their content, produce a larger late positive wave than neutral pictures. As we know, sex-related stimuli contain more biology information than love-related pictures and neural pictures, which are crucial for human’s survival and reproduction. Whether in early or late processing stages, sex-related stimuli always catch more attention and need more processing materials in our brain.

In our study, we tested the mediation effect of adult attachment on individuals’ processing of stimuli from different categories on ERP components (N1, N2, and PSW). Results showed that adult attachment styles were related to recognition of difference stimuli (SEX or LOVE) especially a frontal site on N1 and N2 ERP components. Further analyses showed that voltages elicited by secure attachment style individuals were significant larger than voltage elicited by fearful and preoccupied attachment style individuals on SEX-MINUS-SPORT at frontal sites, on N1 and N2 components. Voltages elicited by dismissing attachment style individuals were larger than voltages elicited by fearful attachment style individuals on SEX-MINUS-SPORT wave at frontal sites, on N2 components. Compared to individuals of the other three adult attachment styles, fearful attachment style individuals are usually frightened and anxious during sexual relationship, which explains the decreased response to sex-related image-stimuli. These results are consistent with results of previous behavior studies that showed that individuals with fearful attachment style engage less in sexual behaviors (Russell and McNulty, 2011), express disgust toward sexual experiences and find it hard to enjoy sexual life (Lambert et al., 2003).

In addition, adult attachment styles may influence level of obsessive love, sexual attitude and sexual experiences in common romantic relationships (Hazan and Shaver, 1987; Kirkpatrick and Hazan, 1994; Feeney et al., 2000; Gentzler and Kerns, 2004). Importantly, individuals with dismissing attachment style have been found to be cold and less expressive (Bartholomew and Horowitz, 1991). In present study, the electrophysiological voltage differences in adult attachment styles were significantly related to recognition of sex-related images while behavior data found that adult attachment styles were not significantly related to any dimension of sexual attitudes. The difference in results between behavior data and electrophysiological data helps to prove that implicit ERP technology results may not be the same with explicit questionnaire behavior data. However, Chinese people’s view of sex is relatively conservative, so self-reported behavior questionnaire method may have many weaknesses for such variables, as individuals tend to report the more reasonable and socially desirable responses. Thus, compared to behavior method, ERP technology can be used widely in future studies to investigate the most spontaneous and real potential reactions of the human brain.

One limitation of the present study merits consideration. In order of presentation of stimuli, in the LOVE block; stimuli were presented in FRIEND-NEUTRAL-LOVE, order while in the SEX block; stimuli were presented in SPORT-NEUTRAL-SEX, order. The fixed order may have affected participants’ attention to different image-stimuli. Future studies should balance the order to capture full attention of participants during the experiment.

## Conclusion

From electrophysiological data, the current study found that response to love-related image-stimuli and sex-related image-stimuli were significantly different on N1, N2, and PSW components. Our results suggest that different adult attachment styles may affect one’s recognition processing in response to love-related images and sex-related images, especially, to sex-related images. Further, from behavior data, we found that adult attachment styles were not significantly related to any dimension of sexual attitudes but showed significant difference in PLS total points. These behavior results were not in line with electrophysiological results. The present study provides evidence that during development of sexual relationship for mating, reproduction, and parenting, there are three primary emotion-motivation systems in human brains, called lust, attraction, and attachment. Using electrophysiological technology, our study also found that adult attachment styles may influence human lust and attraction systems.

## Author Contributions

Conceived and designed the experiments: JH, XC, JL, FY, JNH, and XF. Performed the experiments: JH, XC, JL, FY, and JNH. Analyzed the data: JH, XC, JL, FY, JNH, RM, YZ, JL, and LL. Contributed reagents/materials/analysis tools: JH, XC, JL, FY, JNH, RM, YZ, JL, and LL. Wrote the paper: JH, XC, YN, RM, YZ, and XF. Discussed the result: JH, ZH, YN, RM, YZ, JL, LL, and XF. Final approval of the version to be published: JH, YN, JL, LL, and XF.

## Conflict of Interest Statement

The authors declare that the research was conducted in the absence of any commercial or financial relationships that could be construed as a potential conflict of interest.
